# The Dopaminergic System in Peripheral Blood Lymphocytes: From Physiology to Pharmacology and Potential Applications to Neuropsychiatric Disorders

**DOI:** 10.2174/157015911795596612

**Published:** 2011-06

**Authors:** Francesca R Buttarelli, Alessandra Fanciulli, Clelia Pellicano, Francesco E Pontieri

**Affiliations:** 1 Department of Neurology and Psychiatry, “Sapienza” University; 2 Movement Disorder Unit, “Sant'Andrea” Hospital, Rome, Italy

**Keywords:** Dopamine, dopamine receptors, dopamine transporter, neuropsychiatric disorders, peripheral blood lymphocytes.

## Abstract

Besides its action on the nervous system, dopamine (DA) plays a role on neural-immune interactions. Here we review the current evidence on the dopaminergic system in human peripheral blood lymphocytes (PBL). PBL synthesize DA through the tyrosine-hydroxylase/DOPA-decarboxylase pathway, and express DA receptors and DA transporter (DAT) on their plasma membrane. Stimulation of DA receptors on PBL membrane contributes to modulate the development and initiation of immune responses under physiological conditions and in immune system pathologies such as autoimmunity or immunodeficiency.

The characterization of DA system in PBL gave rise to a further line of research investigating the feasibility of PBL as a cellular model for studying DA derangement in neuropsychiatric disorders. Several reports showed changes of the expression of DAT and/or DA receptors in PBL from patients suffering from several neuropsychiatric disorders, in particular parkinsonian syndromes, schizophrenia and drug- or alcohol-abuse. Despite some methodological and theoretical limitations, these findings suggest that PBL may prove a cellular tool with which to identify the derangement of DA transmission in neuropsychiatric diseases, as well as to monitor the effects of pharmacological treatments.

## INTRODUCTION

The catecholamine, dopamine (DA), plays a cardinal role in the control of motor, cognitive, behavioral and endocrine functions in the central nervous system (CNS) [1]. DA cell bodies in the CNS are located within the substantia nigra pars compacta and ventral tegmental area in the mesencephalon [2]. These neurons may be identified by the positive immunostaining for tyrosine-hydroxylase (TH) and DOPA-decarboxylase [3]. The projecting axons reach the dorsal (nigrostriatal pathway) and ventral (mesolimbic pathway) striatum together with prefrontal cortical areas (mesocortical pathway), respectively [4]. A further DA pathway, defined as tuberoinfundibular, arises from cell bodies in the hypothalamus and reaches the anterior pituitary to inhibit prolactin secretion [5].

The effects of DA in the CNS are mediated through activation of DA receptors. The amino acid sequence of each DA receptor subtype encodes the seven membrane-spanning regions characteristic of G protein-coupled receptors [1, 6]. Two distinct families of DA receptors have been identified, with opposite actions on adenylate cyclase [1, 7]: the D1-like receptors (D1 and D5 receptors) are coupled to a G protein that activates adenylate cyclase, whereas the D2-like receptors (D2, D3 and D4 receptors) are negatively coupled to adenylate cyclase. Moreover, the diverse DA receptor subtypes show different topographic segregation within the CNS [1, 8]: thus, D1 receptors are abundant in the basal ganglia, nucleus accumbens and cerebral cortex, D2 receptors have highest concentrations in the basal ganglia and anterior pituitary, D3 receptors in the ventral striatum (nucleus accumbens, islands of Calleja, olfactory tubercle), D4 receptors show highest density in the frontal cortex, hippocampus and amygdala, and D5 receptors are mainly located in the hippocampus and thalamus.

DA transmission in the CNS is regulated by the level of transmitter available for interactions with DA receptors. This, in turn, depends on the amount of DA released by axonal firing and the rate of metabolism and re-uptake of the transmitter. DA metabolism occurs through two distinct, although partially interacting, pathways, the monoamine-oxidase and the catechol-O-methyl-transferase [1, 9]. Under physiological conditions, DA re-uptake mostly depends on the presence and activity of DA transporter (DAT), a 80 kD glycoprotein belonging to the large Na^+^/Cl^-^ dependent transporter family, which includes norepinephrine, serotonin, GABA and glycine transporters. It consists of a 620-amino acid protein, organized in 12 transmembrane domains, with cytoplasmic amino- and carboxy-termini [10]. Intracellular DA levels available for synaptic transmission are also regulated by the vesicular monoamine transporters (VMAT-1 and VMAT-2) that concentrate the DA into presynaptic vesicles [11].

Neuroanatomical and/or functional derangement of of DA transmission in the CNS is a core feature of several neurological or psychiatric disorders.

The cardinal motor symptoms of Parkinson's disease (PD) depend upon progressive degeneration of DA-containing neurons in the substantia nigra pars compacta and, consequently, reduced extracellular DA concentrations in the striatum [12]. There is also evidence for damage of mesocorticolimbic DA neurons in PD [13]. Moreover, damage of central DA neurons is commonly found in atypical parkinsonisms, including multiple system atrophy (MSA), progressive supranuclear palsy, Lewy bodies dementia and corticobasal degeneration [14], and may be observed in a discrete proportion of subjects suffering vascular or toxic parkinsonism [15]. Blockade of DA receptors is responsible for development of neuroleptic drug-induced parkinsonism [16]. Within movement disorders, finally, there is pharmacological [17] and neuroimaging [18] evidence for the pathological involvement of DA transmission in Tourette's syndrome, a neuropsychiatric disorder characterized by multiple motor tics plus one or more vocal tics.

Alzheimer's disease (AD) is the most common cause of mental deterioration in elderly people. Neocortical deficit of choline acetyltransferase, reduction of choline uptake and acetylcholine release, as well as loss of cholinergic neurons from the nucleus basalis of Meynert, together with the established role of acetylcholine in learning and memory, led to the cholinergic hypothesis of AD [19]. Other neurotransmitter-receptor systems are, however, damaged in AD, including catecholamines. In particular, loss of norepinephrine neurons in the locus coeruleus, degeneration of norepinephrine projections to the basal forebrain, and decreased cortical norepinephrine have been reported in AD [20]. Moreover, AD patients may show motor symptoms suggestive of pathological involvement of the basal ganglia circuitries [12], and previous studies indicate the crucial role of corticostriatal dopaminergic networks in cognitive and motor processes in AD [21]. There is also evidence on the implication of DA in the development of delusions and apathy in AD [22].

Following the first clinical and pharmacological suggestions of altered DA transmission in migraineurs, studies on DA receptors have further emphasized the link between migraine and DA, and suggested that DA receptors in migraineurs have peculiar functional and genetic features, including such as lowered activation threshold [23]. Furthermore, the positive association between allele 1 of D2 receptors and the subgroup of migraineurs presenting both nausea and yawing, two known DA-related phenomena, immediately before or during the pain phase of migraine has been identified [24]. Finally, there is still debate on the possibility that a specific D2 receptor polymorphism might increase susceptibility to migraine with aura [25].

Schizophrenia is one of the most common mental disorders, but its etiology and pathophysiology are still obscure. Several studies have suggested changes in DA systems in schizophrenia. However, the dopaminergic hypothesis for schizophrenia is still largely based on the consequences of pharmacologic manipulations of DA transmission either by mimicking [26] or reducing [27] the symptoms of schizophrenia. With this respect, one should consider that functional changes of DA systems may occur as the consequence of DA itself as well as DA receptors.

Stimulation of mesolimbic DA transmission is a common consequence of the administration of drugs of abuse belonging to different pharmacological classes to experimental animals [28-30] as well as humans [31]. Mesolimbic DA transmission is thought to play a fundamental role for the rewarding properties of these drugs, as well as for the acquisition and maintenance of associative learning processes related to drug addiction and relapse [32].

Finally, DA transmission in the CNS has been implicated in some behavioral features of depression. The strongest evidence implicating DA involvement in depression derived from the observation of lower homovanillic acid concentrations in the cerebrospinal fluid from depressed patients [33,  34]. In particular, the anhedonic symptoms frequently encountered in depressed subjects have been related to altered mesolimbic DA transmission [35] and may be ameliorated by dopaminergic therapies [36]. Conversely, DA receptor antagonists may aggravate depression-like symptoms [35].

Besides of its action on the nervous system, DA has been identified in other organs and tissues, including the vascular beds, the hearth, the gastrointestinal tract, and the kidney. Moreover, a number of studies showed DA components in the immune system, and suggested that DA plays a key role on neural-immune interactions and immune cells in particular. Here we review these articles with the aim to provide an up-to-date definition of the DA system in peripheral blood lymphocytes (PBL), as well as to support the feasibility of PBL as a cellular tool with which to investigate DA derangement in neuropsychiatric disorders. For our purpose, the database was selected using PubMed Services including the following keywords: dopamine, dopamine receptors, dopamine transporter, peripheral blood lymphocytes. In addition, the bibliographies of all relevant articles were searched for further publications. The articles were restricted to English language and spanned the period from January 1980 to February 2010. Historically remarkable or conceptually related articles were included as well. All articles cited in this manuscript were judged by F.R.B. and F.E.P. to be relevant and to meet the scientific and conceptual criteria listed.

## THE DOPAMINERGIC SYSTEM IN PERIPHERAL BLOOD LYMPHOCYTES

The original discovery of endogenous DA in PBL was made by Bergquist *et al.* [37] back in 1994 (Table **1**). The authors applied capillary electrophoresis with electrochemical detection to quantify DA and its metabolites in single lymphocytes and extracts of T- and B-cell clones. Moreover, pharmacological inhibition of TH by α-methyl-p-tyrosine reduced observed catecholamine levels, suggesting direct synthesis of catecholamines by PBL, and intracellular DA levels were increased by exposure to extracellular DA, suggesting the presence of an active cellular uptake mechanism. Four years later [38], the same research group confirmed the presence of DA, L-DOPA, and norepinephrine in PBL by electrospray ionization mass spectroscopy. In 1996, Musso *et al.* [39] studied catecholamine content in human PBL and the ability of these cells to synthesize catecholamines *in vitro*. Catecholamines were separated by high performance liquid chromatography (HPLC) and electrochemical detection. T-lymphocytes contained L-DOPA and norepinephrine, whereas B-lymphocytes contained only L-DOPA. PBL were able to synthesize norepinephrine from both L-tyrosine and L-DOPA added to the incubation medium, thus indicating the presence of active synthetic pathways. In 2004, Qiu *et al.* [40] confirmed that human PBL may synthesize catecholamines, including DA, by combining immunochemical methods to investigate the expression of TH, HPLC to measure catecholamine content, and western blot to examine and quantify TH-stained protein. Moreover, Cosentino *et al*. [41] showed that human T-lymphocytes constitutively express TH, the rate-limiting enzyme in the synthesis of catecholamines, and contain substantial amounts of DA, epinephrine and norepinephrine, which may be released upon treatment with reserpine. Finally, Kikkonou *et al*. [42] reported in 2007 the expression of the gene coding for L-DOPA-decarboxylase in human PBL.

Despite such converging evidence for the presence of catecholamines, and DA in particular, in PBL, early research on the characterization of DA receptors gave rather contradictory results. Studies by Maloteaux *et al*. [43], Fleminger *et al.* [44], Feenstra *et al.* [45], and Coccini *et al.* [46] questioned the presence of DA receptors in PBL. Indeed, these authors showed that haloperidol displaceable component of [^3^H]-spiperone binding to human PBL was not saturable, and that stereoselective displacement by the isomers of butaclamol was not observed. Also, there was no correlation between the ability of known DA active drugs to cause displacement of the ligand and their rank order of potency, thus suggesting that the apparent association of [^3^H]-spiperone with PBL might be due to some passive uptake process causing accumulation of the ligand within the cells. As discussed by Wodarz *et al.* [47], however, methodological biases (unspecific filter binding, which increased the presence of butaclamol, or variable amount of contaminating granulocytes) might have contributed to these negative results.

In 1991, Faraj *et al.* [48] (Table **1**) first reported the occurrence of binding to DA receptors in PBL, markedly affected by cocaine and other inhibitors of biogenic amine uptake. One year later, Takahashi *et al.* [49] applied sequential reverse transcription and polymerase chain reaction (RT-PCR) to demonstrate the presence of three types of mRNA sequences, each corresponding to those of the D5 receptor gene and the two related pseudogenes, in human PBL. The PBL cDNA library also contained the clones encoding parts of the three genes. Binding profiles of dopaminergic ligands to the PBL were similar to those for the native neuronal membranes. Using the same methodologies, Nagai *et al.* [50] reported in 1993 the occurrence of a novel shorter variant transcript of the D3 receptor gene generated by alternative splicing in PBL and brain. Receptor binding studies by Santambrogio *et al.* [51] further contributed to characterize DA receptors in PBL by showing high affinity, specific, saturable and reversible binding of [^3^H]-sulpiride to human PBL. In this latter study, the pharmacological characterization of the binding sites suggested the presence of D2 and D4 receptor subtypes in PBL. In 1994, Ricci and Amenta [52] described the occurrence of D1-like receptors in human PBL by means of radioligand binding technique. In that study, binding to [^3^H]-SCH23390 was applied to localize D1-like receptors. Pharmacological analysis of displacement curves of radioligand with DA competing with the radioligand in submicromolar range suggested the presence of D5 rather than D1 receptors. Using the same technique, these authors also reported the occurrence of high-affinity [^3^H]-OH-DPAT binding to D3 receptors in human PBL [53]. Interestingly, binding was time-, temperature-, and concentration-dependent, and it was also reversible. The rank order of potency of displacers was similar to those found for D3 receptors in rat brain homogenates or in rat or human cell lines. The same research group also showed age-dependent reduction of DA receptor subtypes in human PBL [54]. In 1996, Bondy *et al*. [55] applied RT-PCR to demonstrate the expression of D4 receptors in human PBL. Receptor binding evidence of the expression of this latter subtype of DA receptor was provided the following year by Ricci *et al.* [56]. In 1997, this latter group [57] defined more precisely the occurrence of D3 and D4 receptors by combining receptor binding assay and immunocytochemistry for D2-like receptor subtypes. Within D1-like receptor subtypes, the authors confirmed that only D5 receptors were expressed on PBL membrane [58]. Finally, in 2002, McKenna *et al.* [59] confirmed that D5 receptors were the only D1-like receptors expressed in PBL, whereas all D2-like receptor subtypes (D2, D3, and D4) were expressed. The specificity of expression of D5 receptors was further confirmed by Kirillova *et al.* [60].

The first report of the presence of DAT on human PBL membrane came by Amenta *et al*. in 2001 [61] (Table **1**). In that paper, the authors demonstrated specific binding of [^3^H]-GBR12935 to PBL, with a dissociation constant similar to that found in the striatum, but with lower density of binding sites. Moreover, western blot analysis using antibodies raised against amino- or carboxy-termini of DAT or against VMAT-1 and VMAT-2 revealed labeling of single bands of approximately 76, 55 or 68 kD, respectively, displaying similar migration characteristics in PBL and test tissues used for comparisons. Immunofluorescence revealed that anti-DA, anti-TH, anti-DAT, anti-VMAT-1 and anti-VMAT-2 antibodies labeled the total population of cytospin-centrifugated PBL mounted on microscope slides. Confocal laser microscopy demonstrated that DA and VMAT-2 immunoreactivity was present mainly in cytoplasmic puntiform areas, that were likely to correspond to vesicles, and to a lower extent was associated to plasma membrane. TH immunoreactivity was diffused to cytoplasm and to plasma membrane of PBL, whereas DAT and VMAT-1 immunoreactivities were located almost exclusively in PBL plasma membrane and cytoplasm, respectively. Finally, in 2008, Marazziti *et al.* [62] showed the presence of specific and saturable binding of [^3^H]-WIN35, 428, a very selective DAT binding compound, together with specific [^3^H]-DA re-uptake by human PBL.

## PHYSIOLOGY AND PHARMACOLOGY OF DA SYSTEM IN IMMUNE CELLS

By stimulating DA receptors expressed on PBL membrane, DA from diverse sources (plasma, sympathetic nervous system, autocrine or paracrine secretion by immune cells, CNS) may contribute to regulate the initiation and development of immune responses. 

Studies carried out on human and murine T-cells have shown that stimulation of D1-like receptors impairs T-cell function by causing the rise of intracellular cAMP levels. Further evidence indicates that stimulation of D1-like receptors not only inhibits cytotoxic function of CD8^+^ T-cells [63] but also impairs function and differentiation of T-regulatory cells (Tregs) [41, 64]. Moreover, stimulation of D1-like receptors has been involved in the polarization of naïve CD4^+^ T-cells toward Th17 cells [65, 66]. Because Th17 and Tregs cells are involved in autoimmunity as auto-aggressive and beneficial cells respectively, it is likely that D1-like receptors expressed on T-cells are involved in the interface between autoimmunity and health. Interestingly, the decreased expression of D5 receptors in PBL has been found in patients suffering multiple sclerosis [67].

D2-like receptors are also involved in the modulation of T-cells physiology. For instance, it has been demonstrated that stimulation of these latter receptors promotes enhanced production of interleukin-10, a cytokine that negatively regulates the function of effector T-cells [68]. This inhibition could be involved in the polarization toward Tregs. Regarding D4 receptor stimulation, evidence indicates that this receptor triggers T-cell quiescence by up-regulating Krüppel-like factor-2 (KLF-2) expression [69, 70]. On the other hand, whereas D3-stimulation facilitates differentiation of naïve CD8^+^ T-cells into CTLs [68], it also contributes to polarization of naïve CD4^+^ T-cells toward Th1 effector phenotype [71]. Furthermore, stimulation *via* D3 receptor is thought to be involved in migration and adhesion of T-cells, thus modulating the homing of these cells [71-73].

## CHANGES OF PBL DA SYSTEM IN NEUROLOGICAL DISEASES

Following the initial characterization of the DA system in human PBL, the question raised of whether PBL may represent a useful cellular model with which to investigate the derangement of DA transmission in patients suffering neurological or psychiatric disorders.

Because of the prominent role of DA derangement in parkinsonian syndromes, most studies were centered on these disorders (Table **2**). Early research by Le Fur *et al*. [74] showed the dramatic decrease of the number of [^3^H]-spiroperidol binding sites in PBL from untreated PD patients with respect to controls and patients suffering other neurological disorders. This decrease was linearly correlated with the degree of disability of PD patients, and was rescued by L-DOPA therapy. However, in 1983, Maloteaux *et al.* [75] questioned these results by showing that [^3^H]-spiperone binding to PBL did not reveal the occurrence of DA receptors, although lower values were observed in PD patients and the displaceable binding was increased after L-DOPA treatment, suggesting that the non specific binding was due to trapping presumably in lysosomes. Following these results, the polish group headed by Czlonkowski showed the decrease of [^3^H]-spiroperidol binding to PBL from patients suffering Wilson's disease as compared to blood donors [76, 77]. In 1993, Nagai and collaborators [78] investigated DA receptor mRNAs expression in PBL from 45 PD patients and 21 age-matched controls using RT-PCR method with β-actin as internal control and DA receptor binding. The authors found the statistically significant decrease of the D3 receptor mRNA expression in PBL from PD patients, that correlated with disease severity. Moreover, there was also a decrease of D3 receptor binding sites in PBL from PD patients with respect to controls. Conversely, no change of D5 receptor mRNA expression was detected. Finally, in 1999, Barbanti *et al.* [79] applied receptor binding methods to investigate the changes of D1-like and D2-like receptor sites in PBL from 50 *de novo* PD patients, 36 neurological control subjects (patients suffering essential tremor, MSA and other neurodegenerative diseases) and 26 healthy subjects. In this study, PBL from PD patients showed a higher density of both D1-like and D2-like binding sites that either neurological or healthy control subjects. The pharmacological profile of [^3^H]-SCH23390 and [^3^H]-7OH-DPAT binding was consistent with labeling of D5 and D3 receptor subtypes, respectively. In a subgroup of PD patients, the density of D1-like and D2-like binding sites lowered to values comparable to controls after 3-month therapy with L-DOPA or bromocriptine. The authors suggested that the increased density of D1-like and D2-like receptor on PBL in *de novo* PD patients may represent an up-regulation mechanism resulting from diffuse impairment of DA systems in PD.

Our research group contributed a number of articles on the alterations of DA system in PBL from PD patients. Early studies showed the reduction of intracellular DA concentrations and TH immunoreactivity in PBL from PD patients with respect to healthy subjects [80]. Immunocytochemical methods with semi-quantitative computer-assisted densitometry was also applied to identify the reduction of DAT immunoreactivity in PBL from *de novo* PD patients with respect to healthy controls [81] and patients suffering essential tremor [82], a neurological disorder clinically characterized by postural tremor with slight signs of rigidity that is not accompanied by central DA damage. Despite the reduced expression of DAT on PBL plasma membrane in PD patients, intracellular concentrations of DA were significantly increased by L-DOPA therapy [80, 83], indicating the efficiency of DA re-uptake mechanisms. Finally, in a recent study, we combined immunocytochemistry for DAT on PBL and [^123^I]-fluopane binding to the striatum to investigate the possible correlation between central and peripheral DAT levels in a group of *de novo* PD patients [84]. The results of this latter study showed the lack of significant correlation between PBL and striatal DAT levels. Moreover, there was no correlation between central and peripheral DAT expression in patients suffering essential tremor, whereas there was a highly significant correlation between PBL and striatal DAT expression in PD patients treated with dopaminergic therapy (L-DOPA and/or dopamine agonists) [85].

Further studies showed the reduction of DAT immunoreactivity in PBL also in subjects suffering MSA [86] (Table **3**); in these patients, a slight, not significant, increase of DAT immunoreactivity in PBL was measured following withdrawal from L-DOPA therapy [86]. Finally, the reduction of DAT immunoreactivity was measured also in a subpopulation of subjects suffering amyotrophic lateral sclerosis [87] (Table **3**). With respect to this latter disease, it is relevant to note that pathological [88] and neuroimaging [89] studies showed the partial damage of nigrostriatal DA system.

As to other neurodegenerative diseases (Table **3**), Ferrari *et al.* [90] reported recently the increase of D5 receptor mRNA levels in PBL from patients suffering Tourette's syndrome with respect to healthy subjects. In this study, D5 mRNA expression in PBL showed a highly positive correlation with the severity of compulsive symptoms. There is also initial evidence of the possibility to detect derangement of catecholaminergic systems in PBL from subjects suffering AD. PBL from AD patients showed reduced density of D2-like receptors [91], and increased DOPA-decarboxylase immunoreactivity [92] with respect to those from healthy controls.

Besides of neurodegenerative disorders, the increased density of D3, D4, and D5 receptor binding sites on PBL from patients suffering migraine with respect to healthy subjects has been reported [93, 94] (Table **3**). The authors suggested that this receptor up-regulation might represent a peripheral adaptative response to central DA alterations.

## CHANGES OF PBL DA SYSTEM IN PSYCHIATRIC DISEASES

Studies on the alterations of DA system in PBL from schizophrenic patients (Table **4**) were pointed mostly on the changes of expression of DA receptors. In 1985, Bondy *et al.* [95] reported that specific binding of [^3^H]-spiperone was significantly increased in PBL from unmedicated schizophrenic patients with respect to healthy subjects or unmedicated psychiatric control subjects. However, the author's suggestion of the possibility to apply such method as a vulnerability marker was denied by Griffiths *et al.* [96]. In 2001, Kwak *et al.* [97] applied RT-PCR to investigate the expression of DA receptors in PBL from medicated and unmedicated schizophrenic patients and healthy subjects. D3 receptor mRNA was significantly increased in PBL from unmedicated schizophrenic patients as compared to values in medicated patients and healthy subjects. Conversely, D5 receptor mRNA expression in PBL from unmedicated schizophrenic patients was significantly higher than medicated ones but not healthy subjects. In drug naïve or drug free patients, mRNA for DA receptors peaked after 2 weeks of treatment with antipsychotics, and decreased to levels still above baseline at 8^th^ week of treatment. Moreover, drug naïve and drug free patients were divided into two groups according to DA receptor expression before medication, and the group of patients with increased DA receptor mRNA expression had more severe psychiatric symptoms. In the same year, Ilani *et al*. [98] demonstrated the significant (2- to 7-fold) increase of D3 but not D4 receptor mRNA in PBL from unmedicated schizophrenic patients with respect to healthy subjects. This increase was not affected by treatment with typical or atypical antipsychotic drugs. In 2003, Singh *et al.* [99] reported that the positive antipsychotic effects of loxapine, a mid-potency typical neuroleptic, was associated with reduced D2-like receptor binding in PBL. Finally, in 2006, Boneberg *et al.* [100] investigated the expression of DA receptors in purified human neutrophils, monocytes, B cells, natural killer cells and CD4^+^- and CD8^+^-positive T cells by RT-PCR. The results showed the significant increase of D3 receptor mRNA in T cells and the significant decrease of D4 receptor mRNA expression in CD4^+^-T cells from schizophrenic patients with respect to healthy subjects. In contrast with these reports, Vogel *et al*. [101] in 2004 reported the reduction of D3 receptor mRNA expression in PBL from schizophrenic and bipolar patients. In this study, antipsychotic treatment in schizophrenic patients produced significant increases of D3 receptor mRNA expression. Finally, a recent study by Marazziti *et al*. [102] demonstrated the reduction of DAT expression in PBL from psychotic patients with respect to healthy subjects.

Biermann *et al.* [103] investigated recently the changes of DA receptor expression in PBL during alcohol withdrawal (Table **5**). The increase of D1 receptor expression reached significance in the early phase of withdrawal, whereas a not-significant increase of D2 receptor expression was observed throughout all withdrawal period. Czermak *et al.* [104] reported the reduction of D4 receptor mRNA expression in PBL of long-term abstinent alcohol and heroin addicts, thus suggesting a withdrawal-persisting DA imbalance in abstinent addicts as measured by a suggested peripheral marker. Similarly, Goodarzi *et al.* [105] showed the increase of D3 receptor mRNA expression in PBL from heroin-addicted and methadone-maintained subjects, the decrease of D4 mRNA expression in PBL from heroin-abstinent and heroin-addicted subjects, and the reduction of D5 mRNA expression in heroin-abstinent subjects solely.

As to studies on depression (Table **5**), Rocca *et al*. [106] reported the significant reduction of D4 receptor mRNA in PBL from untreated patients suffering major depression. Such changes were reversed following therapy with paroxetine. The reduction of serotonin, but not DA, turnover in PBL from depressed patients was reported by Fajardo *et al*. [107].

## CONCLUSIONS AND FUTURE PERSPECTIVES

The studies reviewed herein contributed to characterize the dopaminergic system in PBL and to clarify the physiological role of DA on PBL function. Further insight into DA-mediated regulation of immune function is critical to understanding its role in unbalanced immune responses, including autoimmunity, immunodeficiency or tumor growth.

The relevant involvement of DA-mediated regulation of immune response is evidenced by deregulation of DA receptors expressed on T-cells and alterations of plasma DA levels, both conditions found as part of the pathophysiological scenario in some immune-related and neurological disorders. In this regard, deregulation of DA receptors expression and plasma DA levels follow a trend geared toward exacerbate the imbalance of immune response. For instance, plasma DA levels, which in general inhibit T-cell function, are increased in malignancies [108], but decreased in autoimmune disorders [67, 109]. The precise knowledge of deregulation of plasma DA concentration and DA receptors expression on T-cells under different pathophysiological conditions, together with an understanding of the precise role of stimulation of each DA receptor subtype on T-cell physiology could facilitate to the design of therapies for the treatment of autoimmunity, immunodeficiency and cancer.

A further line of research reviewed in the present article dealt with the feasibility of PBL as a cellular model for investigating the alterations of DA system in neuropsychiatric disorders, in particular parkinsonian syndromes and schizophrenia.

With respect to PD (Table **2**), the results from a number of studies demonstrated that PBL may, indeed, represent a tool with which to identify alterations of DA system *in vivo*. Thus, there is evidence for the reduction of intracellular DA content [80], the reduction of TH [80] and DAT [81, 82, 84] immunoreactivities, as well as changes of the expression of DA receptors [78, 79] in PBL from *de novo* PD patients. Such alterations contributed to the definition of the alterations of DA systems outside the brain in the disease [85]. Moreover, the observation that therapy with L-DOPA or DA agonists may reverse the original alterations of the expression of DA receptors [79] suggested that PBL may serve as a cellular tool to investigate *in vivo* the adaptative changes of DA receptors to pharmacological treatments.

The current evidence of reduction of DA markers, and DAT immunoreactivity in particular, in PBL from subjects suffering neurodegenerative disorders (PD, MSA, ALS) [81, 82, 84, 86, 87] (Table **3**), and the observation that such reductions may be measured already in the early stages of these disorders, suggested that alterations of PBL DA system may be identified precociously in these diseases and might, theoretically, contribute to the identification of subjects at risk. Despite such suggestions, the results of recent studies from our group [82, 85, 86] showed the inability for DAT expression in PBL to discriminate between different neurodegenerative disorders involving the central DA systems, and, consequently, the lack of validation of these measurements for the differential diagnosis among these conditions. Moreover, the lack of correlation between striatal and PBL DAT expression in *de novo* PD patients [84] suggested that different mechanisms of regulation of DAT expression occur at central and peripheral level, at least under pathological conditions involving DA-containing cells.

As to other neurodegenerative disorders, changes of DA markers in PBL were measured in Wilson's disease [76], Tourette's syndrome [90], and AD [91] (Table **3**). In particular, the observation of reduced density of D2 receptors in PBL from AD patients [91] suggests to further investigate the possible association between changes of DA receptor density and clinical symptoms such as apathy in the disease. 

As to psychiatric disorders, there is consistent evidence of the possibility to identify changes of the expression of different DA receptor subtypes in PBL from schizophrenic patients [95, 97-101] (Table **4**). In most cases, up-regulation of the expression of D3 receptors was found, suggesting the increased functional response to DA stimulation. Interestingly, these changes appeared to correlate with the severity of psychiatric symptoms [97], and were at least partially modulated by antipsychotic drugs [97, 101]. Thus, these results indicate that PBL may, indeed, represent a useful tool with which to monitor the changes of DA receptor expression with respect to the efficacy of therapeutic interventions. Similarly, dynamic changes of DA receptor subtypes were measured during drug- or alcohol-withdrawal [103-105] (Table **5**). These latter data indirectly confirm the role of DA transmission in the process of drug addiction. Finally, the observations of reduced D4 receptor expression in PBL from depressed patients (Table **5**) gave further support to the pathogenetic role of DA transmission in depression.

Taken together, therefore, the results from a number of studies support the hypothesis that PBL may represent a useful tool for investigating the changes of DA system in CNS pathologies, as well as to monitor with the consequences of pharmacological manipulations of DA transmission. This is, to our opinion, particularly relevant in view of the economical and technical difficulties of investigating such changes directly in the CNS *in vivo*. However, some limitations to the use of PBL as 'surrogate' markers for studying the changes of DA system in neuropsychiatric diseases should be acknowledged, in particular the observation that these cells are surrounded by different environments and, therefore, subjected to different mechanisms of regulation [110]. In any case, the ease and relative safety of obtaining blood cells have promoted *in vivo* studies and provided intriguing information that continues to receive further support.

## Figures and Tables

**Table 1.** The Dopaminergic System in Peripheral Blood Lymphocytes. The Table Summarizes the Methodology Applied and the Findings Obtained. DA=Dopamine, DAT=Dopamine Transporter, VMAT-1, 2= Vesicular Monoamine Transporters

Authors	Methology	Finding
Bergquist *et al.* [37]	Capillary electrophoresis	DA and DA metabolites
Bergquist and Silberring [38]	Mass spectroscopy	DA, L-DOPA, norepinephrine
Musso *et al.* [39]	HPLC with electrochemical detection	L-DOPA and norepinephrine
Qiu *et al. *[40]	Immunochemistry, HPLC, Western blot	Tyrosine hydroxylase, DA, L-DOPA, norepinephrine
Kikkonou *et al*. [42]	RT-PCR	L-DOPA decarboxylase gene
Faraj *et al. *[48]	Receptor binding assay	DA receptors
Takahashi *et al. *[49]	RT-PCR	D5 receptor gene
Nagai *et al. *[50]	RT-PCR	D3 receptor gene
Santambrogio *et al. *[51]	Receptor binding assay	D2, D4 receptors
Ricci and Amenta [52]	Receptor binding assay	D5 receptor
Ricci *et al.* [53]	Receptor binding assay	D3 receptor
Bondy *et al.* [55]	RT-PCR	D4 receptor gene
Ricci *et al.* [56]	Receptor binding assay	D4 receptor
Ricci *et al. *[57]	Receptor binding assay, immunochemistry	D3, D4 receptors
Ricci *et al.* [58]	Receptor binding assay	D5 receptor
McKenna *et al.* [59]	Flow cytometry	D2, D3, D4, D5 receptors
Kirillova *et al.* [60]	Receptor binding assay, RT-PCR	D5 receptor
Amenta *et al.* [61]	Receptor binding assay, Western blot	DAT, VMAT-1, VMAT-2
Marazziti *et al.* [62]	Receptor binding assay	DAT

**Table 2.** Changes of Dopaminergic Markers in Peripheral Blood Lymphocytes in Parkinson's Disease. The Table Summarizes the Methodology Applied and the Findings Obtained. DA=Dopamine, DAT=Dopamine Transporter

Authors	Methodology	Findings
Le Fur *et al.* [74]	Receptor binding assay	Decreased D2-like receptor
Nagai *et al. *[78]	RT-PCR and Receptor binding assay	Decreased D3 receptor
Barbanti *et al.* [79]	Receptor binding assay	Increased D3 and D5 receptors
Caronti *et al.* [80]	Immunochemistry, HPLC with electrochemical detection	Decreased TH immunoreativity, Decreased intracellular DA concentration
Caronti *et al.* [81]	Immunochemistry	Decreased DAT immunoreactivity
Pellicano *et al.* [82]	Immunochemistry	Decreased DAT immunoreactivity
Buttarelli *et al.* [84]	Immunochemistry	Decreased DAT immunoreactivity
Pontieri and Colosimo [85]	Immunochemistry	Decreased DAT immunoreactivity

**Table 3.** Changes of Dopaminergic Markers in Peripheral Blood Lymphocytes in other Neurological Disorders. The Table Summarizes the Methodology Applied and the Findings Obtained. DA=Dopamine, DAT=Dopamine Transporter, DBH=Dopamine-Beta-Hydroxylase

Authors	Methodology	Disease	Findings
Czlonkowski *et al.* [76]	Receptor binding assay	Wilson's disease	Decreased D2-like receptor
	Receptor binding assay	Wilson's disease	Decreased D2-like binding
Buttarelli *et al.* [86]	Immunochemistry	Multiple system atrophy	Decreased DAT immunoreactivity
Buttarelli *et al.* [87]	Immunochemistry	Amyotrophic lateral sclerosis	Decreased DAT immunoreactivity
Ferrari *et al.* [90]	Receptor binding assay	Tourette's syndrome	Increased D5 receptor
Barbanti *et al.* [91]	Receptor binding assay	Alzheimer's disease	Decreased D2-like receptors
Giubilei *et al.* [92]	Immunochemistry	Alzheimer's disease	Increased DBH immunoreactivity
Barbanti *et al.* [93]	Receptor binding assay	Migraine	Increased D5 receptors
Barbanti *et al.* [94]	Receptor binding assay	Migraine	Increased D3, D4 receptors

**Table 4.** Changes of Dopaminergic Markers in Peripheral Blood Lymphocytes in Schizophrenia. The Table Summarizes the Methodology Applied and the Findings Obtained. DAT=Dopamine Transporter

Authors	Methodology	Findings
Bondy *et al.* [95]	Receptor binding assay	Increased D2-like binding
Kwak *et al.* [97]	RT-PCR	Increased D3 receptor mRNA
Ilani *et al.* [98]	RT-PCR	Increased D3 receptor mRNA
Boneberg *et al.* [100]	RT-PCR	Increased D3 receptor mRNA Decreased D4 receptor mRNA
Vogel *et al.* [101]	RT-PCR	Reduced D3 receptor mRNA
Marazziti *et al.* [102]	Receptor binding assay	Reduced DAT binding

**Table 5.** Changes of Dopaminergic Markers in Peripheral Blood lymphocytes in other Psychiatric Disorders. The Table Summarizes the Methodology Applied and the Findings Obtained

Authors	Methodology	Disease	Findings
Biermann *et al.* [103]	RT-PCR	Alcohol withdrawal	Increased D1 receptor mRNA
Czermak *et al.* [104]	RT-PCR	Alcohol- and heroin withdrawal	Reduced D4 receptor mRNA
Goodarzi *et al.* [105]	RT-PCR	Heroin addiction Heroin abstinence	Increased D3 receptor mRNA Decreased D5 receptor mRNA
Rocca *et al.* [106]	RT-PCR	Depression	Reduced D4 receptor mRNA
Fajardo *et al.* [107]	HPLC	Depression	Reduced intracellular serotonin concentration
